# The Feasibility of the Full and Modified Versions of the Alarm Distress Baby Scale (ADBB) and the Prevalence of Social Withdrawal in Infants in Nepal

**DOI:** 10.3389/fpsyg.2020.02025

**Published:** 2020-08-26

**Authors:** Manjeswori Ulak, Suman Ranjitkar, Merina Shrestha, Hanne C. Braarud, Ram K. Chandyo, Laxman Shrestha, Antoine Guedeney, Tor A. Strand, Ingrid Kvestad

**Affiliations:** ^1^Child Health Research Project, Department of Pediatrics, Institute of Medicine, Tribhuvan University, Kathmandu, Nepal; ^2^Regional Centre for Child and Youth Mental Health and Child Welfare, NORCE Norwegian Research Centre, Bergen, Norway; ^3^Department of Community Medicine, Kathmandu Medical College, Kathmandu, Nepal; ^4^Department of Child and Adolescent Psychiatry, Hospital Bichat-Claude Bernard, Université de Paris, Paris, France; ^5^Centre for International Health, University of Bergen, Bergen, Norway; ^6^Department of Research, Innlandet Hospital Trust, Lillehammer, Norway

**Keywords:** ADBB, social withdrawal, infant, feasibility, Nepal

## Abstract

**Background:**

Sustained social withdrawal in infancy may have organic and nonorganic causes and could hinder normal development. The Alarm Distress Baby (ADBB) scale is a widely validated screening tool of social withdrawal in children 2–24 months. The aim of the current study was to evaluate the full and modified ADBB in Nepalese infants in a community-based study.

**Methods:**

We enrolled 600 infants who were video recorded during a pediatric examination. The 36 infants first enrolled were scored by an expert rater, and the subsequent 64 infants were scored by two trained staff with the full ADBB scale. Of the 600 enrolled infants, 597 videos (including the 100 infants scored with the full ADBB) were scored with the modified ADBB (m-ADBB) scale by the trained staff, with 7% double scoring. We measured the interrater agreement and psychometric properties of both scales.

**Results:**

In the 64 infants scored with the full ADBB by two raters, the concordance correlation coefficients (CCCs) indicated poor interrater agreement. For the m-ADBB, the CCCs were better indicating acceptable agreement between raters. The greatest lower bound (GLB) for reliability coefficient for the full ADBB scored by an expert rater indicated good internal consistency, whereas the GLB coefficient for the m-ADBB indicated poorer internal consistency. The Spearman correlation coefficient between the total scores of the two versions was 0.82 (*P* < 0.001). Among the infants scored with the full ADBB, 25% had a score above cutoff (≥5). Scored with the m-ADBB in the full sample, 11.4% of the infants had a score above the suggested cutoff (≥2). In both versions, children achieved high scores on vocalization.

**Conclusion:**

Our findings suggest that the m-ADBB is an acceptable approach to achieve adequate interrater agreement in a large community-based study in Nepal. Results indicate high prevalence of social withdrawal in this population. There are, however, uncertainties on the internal consistency of the scales in this setting, and the validity of the scales needs to be investigated further. More effective training strategies for administration and additional cultural-specific instructions could be important measures to explore before implementing the scale further in this setting.

## Introduction

Social withdrawal in infants when relating to others is a way to regulate the flow of interactions (Guedeney et al., 2013). While brief withdrawals from these interactions are considered normal, sustained withdrawal characterized by less positive behavior, such as lack of eye contact and smiles, utter sounds, and negative behavior such as self-stimulation, may be a warning sign for both organic and relationship disorders (Guedeney and Fermanian, 2001).

Social withdrawal can have both internal, such as temperamental and genetic, causes and external such as relational, causes. Sustained social withdrawal is, for instance, commonly seen in autism and in children with pervasive developmental disorders (Guedeney et al., 2013), but has also been linked to risk factors in the external environment such as maternal distress and postpartum depression symptoms (Braarud et al., 2013). Social withdrawal was observed in 4-, 8-, and 18-months-old Finnish infants whose parents were less interactive with their infants because of poor mental health (Mäntymaa et al., 2008). There are also studies demonstrating that premature birth and low birth weight are associated with social withdrawal at 6 (Braarud et al., 2013) and 12 months (Guedeney et al., 2012). Social-withdrawal behavior has been described in infants at risk of failure to thrive (Guedeney, 2007). In these infants, the insufficient growth is attributed to both organic causes, such as acute and chronic disorders, and nonorganic causes, which could be due to environmental influences, stimulation deprivation, and poor feeding techniques (Ross et al., 2017).

In the last decades, there has been an increased interest in early child development in low to middle income countries, that is, that a large proportion of young children from low-resource settings fail to reach the expected developmental milestones during their early childhood (Black et al., 2017). This has been attributed to the lack of nurturing care defined as “a stable environment that is sensitive to children’s health and nutritional needs, with protection from threats, opportunities for early learning, and interactions that are responsive, emotionally supportive, and developmentally stimulating” (Britto et al., 2016). In Nepal, which is among the least developed countries in the world with one-third of the population living below the poverty line (CBS, 2018), the life of vulnerable children is characterized by risks such as high degree of infections (MOHN, 2016), micronutrient deficiencies (Ulak et al., 2016), and lack of adequate stimulation and learning opportunities in their home environment (Shrestha et al., 2019). Sustained social withdrawal in infancy, independent of origin, may hinder normal child development through disturbances in social interaction with others (Guedeney et al., 2013). In this perspective, screening of social withdrawal could be beneficial for early detection of nonoptimal development in Nepalese infants.

The detection of social withdrawal may be challenging, however, and require skills and knowledge in infant mental health. A brief screening instrument can facilitate a more structured observation of the infant’s social behavior. In this context, the Alarm Distress Baby (ADBB) scale could be a relevant tool to assess withdrawal in infants 2–24 months of age (Guedeney and Fermanian, 2001). The scale is constructed to assess an infant’s social behavior during interaction with an unfamiliar person, such as a doctor, nurse, psychologist, or other health professionals during routine consultations.

The full ADBB scale examines social withdrawal through eight domains: facial expression, eye contact, general level of activity, self-stimulating gestures, vocalizations, briskness of response to stimulation, relationship with the observer, and the capacity to attract and maintain attention with the observer. Validity and reliability studies of the scale have shown good results (Guedeney et al., 2013). The face validity of the ADBB scale has been evidenced in many studies and across several countries (Guedeney et al., 2012; Smith-Nielsen et al., 2018), as well as in public health centers (Puura et al., 2010). In a study in well-baby clinics in Brazil, the interrater agreement was good, but the agreement was significantly higher between pediatricians [intraclass correlation coefficient (ICC) = 0.82] than between nurses (ICC = 0.61) (Lopes et al., 2008). The prevalence of social withdrawal at 12 months in a large prospective birth cohort study in France was 14% (Guedeney et al., 2012), whereas in a clinical population of human immunodeficiency virus–infected mother–infant pairs in South Africa, 31% of the infants were classified as socially withdrawn (Hartley et al., 2010). In the previous French cohort, scores on the ADBB scale at 1 year were associated with language and motor scores at the same age (Guedeney et al., 2016) and general ability (IQ) scores at 5–6 years (Guedeney et al., 2017).

Following a study on the ADBB scale in clinical practice in Australia, a modified version of the scale has been suggested. In this version, items of the full version that were highly correlated with other items were combined (i.e., briskness of response to stimulation and general level of activity) or removed (i.e., the capacity to attract and maintain attention with the observer). Items that were difficult to score were also removed (i.e., self-stimulating gestures) (Matthey et al., 2013). Hence, the modified ADBB (m-ADBB) examines social withdrawal through only five domains: facial expression, eye contact, vocalization, general level of activity, and relationship with the observer. The scale has a simpler mode of scoring than the full version with fewer response categories. The m-ADBB is straightforward and comprehendible, and it has been demonstrated that for both clinicians and researcher, interrater agreement can be easily reached through this version (Guedeney et al., 2013).

To our knowledge, there are no studies on social withdrawal in infants in South Asian countries, and thus, the feasibility of the scale is unknown in a Nepalese setting. In a large community-based clinical trial in Bhaktapur, Nepal, we enrolled 600 infants 6–11 months from April 2015 to February 2017 (Strand et al., 2017). Bhaktapur municipality is situated east of the capital Kathmandu and, as per the last census conducted in 2011, one of the most densely populated municipalities in Nepal where more than 80,000 people reside in 7 km^2^. During the enrollment procedures of the community-based trial, we made video films of the 600 infants and scored these with the full and modified versions of the ADBB scale. The main aim of the current study is to evaluate the feasibility of both versions of the scale in a large community setting with Nepalese infants. A second goal is to describe the prevalence of social-withdrawal behavior within Nepalese infants, using both the full and modified versions of the ADBB. Finally, the third goal of the study is to describe the profile of social-withdrawal behaviors of the Nepalese infants, as this is the first study of both scales in Asia.

## Materials and Methods

### Study Setting and Participants

The study is part of a large population-based randomized placebo-controlled trial assessing the effect of vitamin B_12_ supplementation on infant’s growth and development. A total of 600 infants aged 6–11 months at enrolment were included in the original study. Field workers prescreened infants from the community and a nearby immunization clinic, and study supervisors or a physician screened the children for eligibility at the study clinic. We included mild to moderate stunted children (length for age <−1*z* score), with plans to reside in the study area or surrounding areas for at least 2 years, in which the caregiver consented for participation. Exclusion criteria were severe malnutrition, severe anemia (hemoglobin < 7 g/dL), systemic chronic illness, taking vitamin supplementation containing vitamin B_12_ in the last month, and any ongoing acute infections such as fever, diarrhea, and/or acute respiratory infection. Details of the original study are described elsewhere (Strand et al., 2017; Chandyo et al., 2018). The mean age of the 600 enrolled infants was 8 months, and 50% were males. The mean birth weight was 2,787 g, with one in every five born with low birth weight (<2,500 g). Most of the mothers (62%) were housewives or worked in agriculture, and 44% have completed school at the level of high school and above. More than two-thirds of the participating families were from the Newar ethnic group. Most of the infants were still breastfed at the time of enrollment. One-third of the infants were stunted (≤2 *z* score), and almost three-fourths of the infants had anemia after adjusting for the local altitude (hemoglobin < 113 g/L).

The study was approved by the Nepal Health Research Council (Reg 233/2014) and the Norwegian Regional Committee for Medical and Health Research Ethics (REC # 2014/1528). All participating parents signed a written informed consent form prior to enrollment and the video recording.

### The Alarm Distress Baby Scale

The ADBB scale is an observational tool to assess sustained social withdrawal in young children between 2 and 24 months of age. A trained examiner needs an observation of 10–15 min to score the ADBB. The full ADBB scale includes eight items (Table 1). Each item is given a score of 0–4: absolutely normal behavior (0), slight abnormal behavior (1), clearly abnormal behavior (2), frankly abnormal behavior (3), and massively obvious abnormal behavior (4). The total possible score of the full ADBB is 32 with a minimum score of 0, and a higher score indicates that the infant displays more social-withdrawal behavior. The cutoff score is suggested to be greater than or equal to 5 based on studies in French infants (Guedeney et al., 2013).

**TABLE 1:** Overview of the full and modified Alarm Distress Baby (ADBB) scale.

Full ADBB^a^	Modified ADBB^b^
1. Facial expression	1. Facial expression
2. Eye contact	2. Eye contact
3. General level of activity	3. General level of activity
4. Self-stimulation	
5. Vocalization	4. Vocalization
6. Briskness of response to stimulation	
7. Relationship to observer	5. Relationship to observer
8. Attraction	

**^*a*^Items scored 0–4, possible score range from 0 to 32, cutoff ≥ 5. ^*b*^Items scored 0 to 2, except vocalization, which is scored 0 to 1; possible scores ranged from 0 to 9, cutoff ≥ 2.**

The m-ADBB is a shorter version of the ADBB scale that includes only five items (Table 1). The items of the m-ADBB are rated with a three-point scoring format: satisfactory (0), possible problem (1) and definite problem (2), with the exception of the vocalization item, which is scored satisfactory (0) or possible problem (1) due to the fact that many infants may be quiet in an unfamiliar setting (Matthey et al., 2013). The scale is rated whenever the participating infant demonstrates his/her behavior with the caregiver or observer throughout the screening period, except for eye contact and relationship, which are rated only with the infant’s behavior toward the examiner. The total score of the m-ADBB is 9, with higher scores indicating more social-withdrawal behavior. The cutoff score is suggested to be greater than or equal to 2 based on studies in an Australian sample of infants (Matthey et al., 2013).

For both versions, a manual provides detailed instructions on the scoring procedures for each item. The scoring is generic and similar for all children within the age range of 2–24 months. Knowledge in normal development in young children is a prerequisite for ADBB raters.

### Training, Standardization, and Certification

Prior to the study, four study staffs including two psychologists (IK and SR) and two pediatricians (MU and MS) participated in a 3-day hands-on training on the full ADBB and m-ADBB with the developer of the ADBB scale (AG). The training was followed by standardization exercises with an expert rater (HCB). First, the staff was given a set of nine training videos to rate individually. Feedback and comparisons with reference scores from the developer of the scale (AG) were given through supervisions with the expert rater. When each staff member scored the same as the reference score on caseness (below/above cutoff for social withdrawal, i.e., a score *≥* 5), the standardization process continued with scoring of infants enrolled in the Nepalese study, starting with the child who was first enrolled. Video recordings were scored individually by the staff and the expert rater. The supervision continued with the expert rater, but this time with the expert’s scores as reference scores. When acceptable agreement was reached, 10 consecutive enrollment videos with reference scores from the expert rater were used for certification. All raters scored the same infants, and scorings were compared with the scores of the expert rater to evaluate the reliability of the study staff. From the standardization and certification procedures, we have full ADBB scorings in the 36 infants who were first enrolled in the study, scored with reference scores from the expert rater.

**FIGURE 1:**
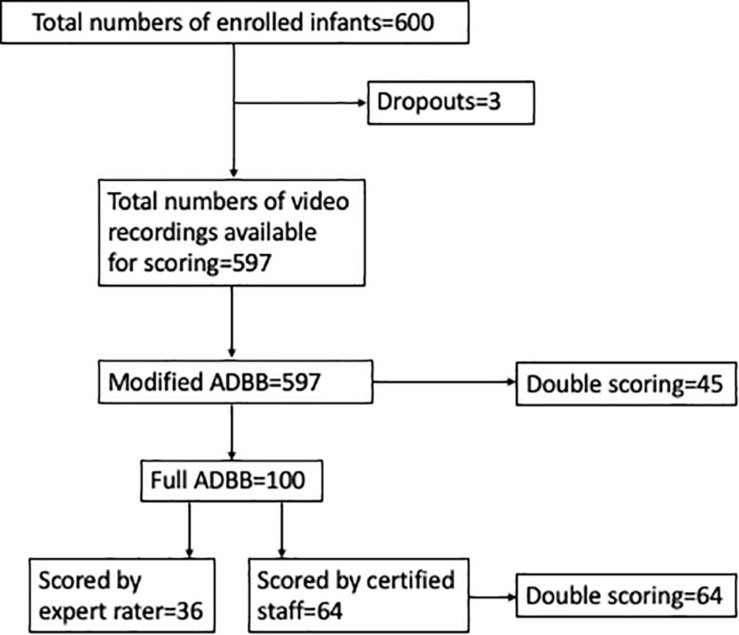
Flowchart of the 600 enrolled Nepalese infants and the number of modified and full ADBB scorings in the study.

Then, the trained and certified staff scored the consecutive enrolled 64 infants with the full ADBB scale, where each infant was scored by two raters individually. Because of poor interrater agreement between the newly certified staff in these scorings, we decided to score the total sample of 600 infants with the m-ADBB version. These 600 infants also include the 100 infants first enrolled to the study, previously scored with the full ADBB. In this process, after a refreshment workshop, the videos were scored one-by-one by three raters, with double scorings in 45 infants (7.5%) by the fourth rater (Figure 1). All the scores were done independently, and the final scores were submitted confidentially. The expert rater was not involved in the m-ADBB scorings.

### Procedure

Infants were video recorded in the enrolment procedure during interaction with a study supervisor or physician in the presence of a caregiver. During this procedure, the caregiver received information on the study, and infant length and weight were taken. During the examination, infants were engaged in social interactions with the examiner. To make the assessment as uniform as possible, we had guidelines on how to structure the situation. It was made sure that the infants were well fed and not sick and sleepy. We used the same well-lit room for all assessments where the examiners were seated in the same position in the eye level of the child. The number of staff present was kept as low as possible during the video recordings, and the duration of the films was kept to more than 10 min.

### Statistical Analyses

Categorical variables are described by frequencies (number) and percentages, and the continuous variables by mean (standard deviation) and range. To estimate the interrater agreement, we used the concord command in Stata to calculate the concordance correlation coefficients (CCCs) between raters for the full ADBB and m-ADBB (Lawrence and Lin, 1989). The CCCs between the trained and certified staff when scoring the full ADBB in 64 infants were poor, and we used only the scores of the 36 infants scored by the expert rater in the further analyses. Because of the skewed distribution of the items, we measured the internal consistency of the scales by the greatest lower bound (GLB) for reliability coefficient (Jackson and Agunwamba, 1977; Trizano-Hermosilla and Alvarado, 2016). The GLB has been considered a better measure of internal consistency than the Cronbach α, which has been criticized for underestimating reliability and not incorporating measurement errors (Sijtsma, 2009; Trizano-Hermosilla and Alvarado, 2016). Spearman correlation was used to estimate correlations between the total and item scores in each version and also between the five comparable items of the full ADBB and m-ADBB (i.e., facial expression, eye contact, general level of activity, vocalization, and relationship). Data were analyzed using Stata version 16 (Stata, College Station, TX, United States) and JASP (version 0.10.2).

## Results

Of the 600 enrolled participants, three did not have complete video recordings, and thus 597 infants were included for the m-ADBB scoring. We have data from three different scoring conditions: 36 infants were scored with the full ADBB by an expert rater; 64 infants were double-scored with the full ADBB by the four certified staffs; and finally, 597 infants (including the previous 36 and 64 infants) were scored with the m-ADBB individually by three certified staff, with 7.5% (*n* = 45) double scoring from the fourth rater (Figure 1). The demographic and clinical characteristics of the infants are described in Table 2.

**TABLE 2:** Baseline information of 600 Nepalese infants in the Alarm Distress Baby Scale study.

Child characteristics		n (%)/Mean (*SD*)
Age of child in months		8 (±1.7)
Male child		309 (51.5%
Home delivery		23 (4%)
Cesarean section delivery		148 (29.6%)
Mean (SD) birth weight, g		2787 (±497)
Preterm birth (<37 weeks)		62 (10.4%)
Birth order	First	292 (48.7%)
	Second or more	308 (41.3%)
Low birth weight (<2,500 g)		119 (20%)
Mean hemoglobin, g/L (SD)		105.9 (±9.3)
Anemia (hemoglobin < 110 g/L)		408 (68.3%)
Anemia (hemoglobin < 113 g/L)^2^		447 (74.5%)
**Demographic features**		
Mother’s age		27 (±4)
Literacy of mother	Illiterate or up to grade 5	223 (37.2%)
	Grades 5–10	113 (18.8%)
	> 10 grade	264 (44%)
Occupation of mother	No working mother/agriculture	374 (62.1%)
	Daily wage earner	90 (15%)
	Services/self-employed	136 (22.7%)
Occupation of father	No working/agriculture	34 (5.6%)
	Daily wage earner/abroad	278 (46.4%)
	Services/self-employed	288 (47.9%)
Ethnic group	Newar	422 (70.3%)
	Other	178 (29.7%)
Socioeconomic status	Family staying in joint family	292 (48.7%)
	Family residing in rented house	291 (48.5%)
	≤2 rooms in use by the household	2337 (56.2%)
	Kitchen and bedroom same	298 (49.7%)
	Family having own land	282 (47%)
	Remittance from abroad	57 (9.5%)
Drinking water supply	Bottle/jar water	46 (7.6%)
	Tap water/tanker supply	533 (88.8%)
	Well, hand pump, or other	21 (3.6%)
Type of cooking fuel	Firewood/kerosene	113 (18.8%)
	Gas	477 (79.5%)
	Electricity	10 (1.7%)

### Interrater Agreement of the Full and Modified ADBB

We examined the interrater agreement between the certified raters for both the full (*n* = 64) and m-ADBB (*n* = 45) (Table 3). The CCCs for the full ADBB scored in 64 infants were poor for both the individual items and the total score. An exception was the vocalization item in which the CCC indicated an acceptable interrater agreement. For the m-ADBB, the CCCs suggested an agreement ranging from moderate to excellent for three of the items and a good interrater agreement for the total score. The eye contact and general level of activity items had too little variation in the scores for the CCC to be calculated.

**TABLE 3:** Interrater agreement between raters in the full and modified Alarm Distress Baby (ADBB) scale in Nepalese infants.

Item	Full ADBB (*n* = 64)	CCC^a^	Item	Modified ADBB (*n* = 45)	CCC^b^
1	Facial expression	0.19 (−0.03, 0.42)	1	Facial expression	0.55 (0.0.34, 0.75)
2	Eye contact	−0.07 (−0.31, 0.17)	2	Eye contact^c^	—
3	General level of activity	0.10 (−0.14, 0.34)	3	General level of activity^c^	—
4	Self-stimulation	−0.03 (−0.21, 0.15)			
5	Vocalization	0.75 (0.65, 0.86)	4	Vocalization	0.93 (0.88, 0.97)
6	Briskness of response to stimulation	0.00 (0.00, 0.25)			
7	Relationship	0.13 (−0.11, 0.38)	5	Relationship	0.66 (0.50, 0.81)
8	Attraction	0.15 (0.00, 0.39)			
	Total	0.39 (0.18, 0.60)		Total	0.81 (0.71, 0.91)

**^*a*^Concordance correlation coefficient. ^*b*^Between 1st and 2nd scorer. ^*c*^No score due to perfect overlap.**

### Internal Consistency of the Full and Modified ADBB

Tables 4 and 5 show the GLB for reliability coefficients for the total scores of the full (*n* = 36) and modified (*n* = 597) ADBB and the correlations between the total and item scores. The GLB coefficient for the full version scored by the expert rater was 0.74 indicating good internal consistency. In this scale, facial expression, eye contact, vocalization, relationship, and attraction were positively and significantly correlated with the total score. For the m-ADBB, the GLB coefficient indicated poorer internal consistency with a value of 0.46. In this scale, all items were positively and significantly correlated with the total score, although vocalization and facial expression showed the strongest correlations.

**TABLE 4:** Internal consistency of the full Alarm Distress Baby Scale in 36 Nepalese infants.

		n	GLB^a^	Between-item correlations^b^								
					1	2	3	4	5	6	7	8
Items				Total Score	Facial expression	Eye contact	Activity level	Self-stimulation	Vocalization	Briskness of response	Relationship	Attraction
	Total score	36	0.74^c^	–								
1	Facial expression			0.69 (<0.001)	–							
2	Eye contact			0.63 (<0.001)	0.50 (0.002)	–						
3	Activity level			0.30 (0.073)	0.09 (0.592)	0.06 (0.742)	–					
4	Self-stimulation			0.21 (0.216)	0.23 (0.186)	0.34 (0.041)	-0.10 (0.572)	–				
5	Vocalization			0.70 (<0.001)	0.20 (0.241)	0.10 (0.556)	0.24 (0.158)	-0.24 (0.153)	–			
6	Briskness of response			–	–	–	–	–	–	–		
7	Relationship			0.67 (<0.001)	0.53 (0.001)	0.56 (0.001)	0.12 (0.494)	0.13 (0.447)	0.28 (0.095)		–	
8	Attraction			0.75 (<0.001)	0.75 (<0.001)	0.57 (0.001)	0.19 (0.270)	0.19 (0.278)	0.27 (0.101)	–	0.73 (<0.001)	–

**^*a*^Greatest lower bound for reliability coefficient. ^*b*^Spearman ρ. ^*c*^Briskness of response was constant and dropped from the analysis.**

**TABLE 5:** Internal consistency of the modified Alarm Distress Baby Scale in 597 Nepalese infants.

		n	GLB^a^	Between-item correlations^b^					
					1	2	3	4	5
Items				Total score	Facial expression	Eye contact	General level of activity	Vocalization	Relationship
	Total score	597	0.46						
1.	Facial expression			0.56 (<0.001)	—				
2.	Eye contact			0.26 (<0.001)	0.10 (0.018)	—			
3.	General level of activity			0.20 (<0.001)	0.01 (0.824)	0.04 (0.392)	—		
4.	Vocalization			0.82 (<0.001)	0.15 (<0.001)	-0.01 (0.793)	0.06 (0.172)	—	
5.	Relationship			0.27 (<0.001)	0.15 (<0.001)	0.14 (<0.001)	0.21 (<0.001)	0.13 (0.001)	—

**^*a*^Greatest lower bound for reliability coefficient. ^*b*^Spearman ρ.**

### Descriptive Statistics of the Full and Modified ADBB

Table 6 shows the mean (SD), range, and the number (%) of children with scores above cutoff in the full ADBB (*n* = 36) scored by the expert rater. The prevalence of social withdrawal (i.e., scores ≥ 5) using the full ADBB was 25%. The infants received high scores on the vocalization item.

**TABLE 6:** Mean (SD) and range of the Full Alarm Distress Baby (ADBB) scale scores (*n* = 36), % of children in each response category and number (%) of children scoring above cut off for social withdrawal.

Items	Mean (*SD*)	Range	N (%) above cutoff	Scores of the full ADBB				
				No abnormal behavior (score = 0)	Slightly abnormal behavior (score = 1)	Clearly abnormal behavior (score = 2)	Frankly abnormal behavior (score = 3)	Obvious abnormal behavior (score = 4)
Facial expression	0.55 (0.77)	0–3		68%	31%	0.08%	0.02%	0
Eye contact	0.33 (0.47)	0–1		67%	33%	0	0	0
Activity level	0.13 (0.47)	0–1		86%	24%	0	0	0
Self-stimulation	0.08 (0.36)	0–2		94%	1%	1%	0	0
Vocalization	1.55 (1.0)	0–3		25%	13%	42%	20%	0
Briskness of response	0	0–0		75%	19%	6%	0	0
Relationship	0.30 (0.57)	0–2		75%	19%	6%	0	0
Attraction	0.36 (0.68)	0–2		75%	24%	6%	0	0
Total score	0.33 (0.68)	0–10	9 (25%)^a^					

*^*a*^Cutoff; total full ADBB ≥ 5.*

Table 7 shows the mean (SD), range, and the number (%) of children with scores above cutoff in the m-ADBB (*n* = 597). According to this scale, the prevalence of social withdrawal in the Nepalese infants (score ≥ 2) was 11.4% in the total sample of infants. When we included only the 36 infants who also was scored with the full ADBB, the prevalence of social withdrawal scored with the m-ADBB was 19.4% (7 infants). The children also received high scores on the vocalization item in the modified version.

**TABLE 7:** Mean (SD) and range of the Modified Alarm Distress Baby (m-ADBB) scale scores (*n* = 597), % of children in each response category and number (%) of children scoring above cut off for social withdrawal.

Items	Mean (SD)	Range	N (%) above cutoff	Scores of the m-ADBB		
				Satisfactory (score = 0)	Possible problem (score = 1)	Definite problem (score = 2)
Facial expression	0.15 (0.36)	0–2		85.6%	14.2%	0.2%
Eye contact	0.04 (0.21)	0–2		96.4%	3.3%	0.4%
Activity level	0.02 (0.14)	0–1		98%	2%	0%
Vocalization	0.39 (0.49)	0–1		60.8%	39.2%	–
Relationship	0.03 (0.16)	0–1		97.5%	2.5%	0%
Total score	0.63 (0.7)	0–5	68 (11.4%)^a^			

*^*a*^Cutoff; total m-ADBB ≥ 2.*

The correlations between the total and items scores of the full (scored by expert rater) and modified (scored by three certified staff) ADBB for the 36 infants first enrolled in the study are shown in Table 8. Except for the eye contact item, the correlations between the total and item scores of the two versions were highly significant (Table 8).

**TABLE 8:** Correlation between the full and modified ADBB items in 36 Nepalese infants.

Items	Spearman ρ (95% CI)	*P*
Facial expression	0.67 (0.44, 0.82)	<0.001
Eye contact with examiner	0.24 (−0.10, 0.53)	0.160
General level of activity	0.60 (0.34, 0.78)	<0.001
Vocalization	0.89 (0.79, 0.94)	<0.001
Relationship with examiner	0.54 (0.25, 0.74)	<0.001
Total score	0.82 (0.68, 0.91)	< 0.001

## Discussion

This is the first Asian study evaluating the use of the two versions of the ADBB scale for the assessment of social withdrawal in Nepalese infants 6–11 months of age in a community-based study setting. Four raters were certified to perform the ADBB scoring. For the full ADBB scale, the interrater agreement between these raters was poor, whereas the agreement for the modified and simplified version of the ADBB was good. The GLB for reliability coefficient of the full ADBB scale scored by an expert rater in 36 infants indicated that the scale has satisfactory internal consistency. The prevalence of social withdrawal assessed with this version was 25%. The GLB coefficient of the modified version scored by certified raters suggested poorer internal consistency in the full sample of 597 children, and scores suggest a prevalence of social withdrawal of 11.4%. The total scores of the two versions were highly correlated. For both versions, the infants received high scores on vocalization, which means that the study infants vocalized less than what is expected in infants from populations where the ADBB scales were developed.

The certified staff achieved better interrater agreement when using the m-ADBB compared to the full ADBB scale. The m-ADBB was developed as a simplified version of the full scale where items that were difficult to score and highly correlated with each other were removed (Matthey et al., 2013). The number of items in the modified version is five compared to eight in the full version. In addition, the scoring criteria are easier, with only three alternatives in the modified version compared to five in the full. One of the aims of the modified version was to simplify the scoring in order to enhance the interrater agreement (Guedeney et al., 2013; Matthey et al., 2013). In accordance with the current findings, previous studies from Australia and South Africa have shown promising interrater agreement using the m-ADBB (Guedeney et al., 2013; Durandt, 2014).

Interrater agreement is a way of quantifying the degree of agreement between raters demonstrating consistency of the scorings (Hallgren, 2012). Low interrater agreement indicates that there is a large amount of measurement errors adding noise to the measurements. As a result, there is an increased probability of type II errors. Acceptable interrater agreement on measurements is accordingly a prerequisite for high-quality research involving quantifications of observations. There could be many reasons for low interrater agreement in a study, including poor psychometric properties of the scale, poorly trained raters. and difficulty in observing and scoring the behavior of interest (Hallgren, 2012). In contrast to our study, there are previous studies that have shown that it is possible to reach acceptable agreement with the full ADBB scale and moreover that the psychometric properties of the scale are good (Guedeney et al., 2013). Although the raters in the current study received a comprehensive training and standardization in order to be certified raters, the poor interrater agreement could indicate that there were difficulties and unclarities in using the scale to evaluate the social behavior of these Nepalese infants. Cultural differences in the understanding of child development could be part of the explanation, and thus more or perhaps a different training content could be necessary to achieve acceptable interrater agreement in this setting. The comparably better CCCs on the modified version with a lower number of items and scoring alternatives demonstrate that the interrater agreement can be improved when the complexity is reduced. Hence, the m-ADBB scale may be a better choice to achieve reliable measures of infant social withdrawal in the current study setting.

We assessed the internal consistency of both the full and modified versions by calculating the GLB for reliability coefficient and by examining the correlations between the total and item scores within each version. The GLB coefficients suggest good internal consistency of the full version scored by the expert rater, whereas the internal consistency of the modified version is poorer. It should be noted that the GLB coefficient has been criticized for overestimating values and that the risk of overestimation increases with lower sample size and larger numbers of items (Oosterwijk et al., 2016). Hence, we cannot rule out that the higher GLB coefficient of the full version could be explained by a lower sample size (36 infants) and that there are more items (8 items compared to 5) in the calculations and not due to that the internal consistency is better. On the other hand, the comparably higher internal consistency of the full version may be due to the fact that the expert scores were used for this calculation. Using the scores in the 64 infants scored by the trained staff would most likely have yielded scores indicating lower internal consistency of this scale. Cultural differences between professionals in the field of child development from Asian and westernized societies may have implications for the understanding of social behavior in infants. Although following a comprehensive procedure of training and standardization in the current study, the consequences of these cultural differences could be that different and more comprehensive training procedures are needed to achieve reliable and valid ADBB measures in an Asian setting.

In the correlation matrices, we see that, for both scales, vocalization is the item that is most strongly correlated with the total score. For the m-ADBB, this item is followed by facial expression, whereas the remaining items are correlated with the total score to a lesser degree. Accordingly, it seems that in our study it is vocalization and facial expression that determine most of the total m-ADBB score, which could explain the low GLB coefficient of the scale in this setting. The discrepancy may be due to the fact that the vocalization and facial expression items are in our impression, the most objective items to score, and thus the loading on the total score differs from that of the other items. Lack of internal consistency of a scale could question the validity of findings in terms of what the scale is measuring. Taken together, our result suggests that this uncertainty exists for both the full and modified versions of the ADBB in this setting (Oosterwijk et al., 2016).

The strong and statistically significant correlation between the total score of the full and modified versions is an important finding in this context. The correlation demonstrates that when the score of the full version increases, indicating more social withdrawal in the infant, that is also true for the modified version. More studies examining the validity of the scales, for instance, in how the total and subscale scores of both versions are related to other known risk factors for early child development in this population, are warranted.

Our results found a prevalence of social withdrawal of 25% in the 36 infants scored with the full ADBB version and of 11.4% scored with the modified version in 597 infants. Interestingly, the prevalence measured by the m-ADBB increased to 19.4% when the analyses were restricted to the 36 children who were also scored by the full version. This suggests that in these 36 infants the prevalence of social withdrawal was higher than in the total sample of 597 infants. The prevalence of social withdrawal assessed by the full version in the total sample is not known. To the best of our knowledge, the full and modified versions have not been much used in the same sample of children before. We see a marginal difference between the versions in the prevalences in the first enrolled 36 infants. Some of the explanation for this difference could be that because the modified version is a cruder measure, its accuracy is poorer than for the full version. More studies are needed to understand this difference fully.

The prevalence of social withdrawal among these Nepalese infants is high, in particular using the full ADBB in the 36 first enrolled infants, compared to previous studies in other cultural settings. In a French cohort study in 12-month-old children of both full-term and premature infants, the prevalence of social withdrawal was 14% using the full ADBB (Guedeney et al., 2012), which is considerably lower than when we use the full version. The prevalence among moderate preterm infants from Norway was fairly low (3.5%) at 9 months of age (Braarud et al., 2013), whereas the prevalence rates in an Israeli study were 38.9% in a clinical group and 11.6% in a control group (Dollberg et al., 2006). Our study participants, although not from a clinical population, face a range of risk factors for nonoptimal development such as micronutrient deficiencies (Ulak et al., 2016), infectious diseases (MOHN, 2016), and the lack of stimulation and learning opportunities (Shrestha et al., 2019). In the current study, these risks are attenuated by the fact that we were targeting children at risk of stunting (length for age *z* score < −1), which is a well-established risk factor for adverse early child development (Black et al., 2017). Furthermore, children at risk of stunting are more likely to live in families from comparably low socioeconomic settings. Taking these risks into consideration, we would expect higher prevalence of social withdrawal in the current population than in populations from high-income countries. One study from South Africa has used the m-ADBB in 83 mother–infant HIV-infected pairs from a low-resource setting which may be more comparable to the Nepalese setting (Durandt, 2014). In this study, the prevalence of social withdrawal is suggested to be 31% using a cutoff of 2, which is considerably above the prevalence found in the current study. Although a high-risk population, we do not target a clinical population, which may explain some of the differences. There are no previous studies evaluating the ADBB scales in Nepal, and more studies are needed to establish the prevalence of social withdrawal in infants from this setting.

The high scores in the vocalization domain both in the full and modified versions should be noted, indicating low levels of vocal utterances in these Nepalese children. We have recently published findings from the same study children, indicating lower performance than expected on expressive language based on US norms measured by the Bayley Scales of Infant and Toddlers Development, third edition (Ranjitkar et al., 2018). The low levels of vocalization may be due to cross-cultural differences, such as being more reserved in the face of strangers or that caregivers are less talkative with their children in this setting compared to in Western societies. Moreover, children in a Western setting are often used to deal with strangers more frequently, for example, by using daycare facilities. Previous findings from the current population suggest that mothers lack knowledge on child development and that most of the mothers are unaware of age-appropriate simple cognitive stimulations to their children, such as talking to the child, showing colorful toys, or shared reading (Shrestha et al., 2019). A study from a different region of Nepal shows that early child development is associated with the home environment, including the verbal responsiveness of the caregiver (Parajuli et al., 2015). These factors could provide some insight to the low levels of vocalization on the ADBB scorings.

Strengths of the study are the large sample size in a well-conducted community-based trial. The raters (two pediatricians and two psychologists) were trained by the developer of the ADBB scale and an expert rater. Furthermore, the raters are experts in child development and have previous experience in infant assessment, as well as in the systematic thinking required for large research projects. As we enrolled infants at risk of stunting, our sample does not fully reflect a general population, which limits the transferability of our results to the Nepalese population as a whole. Other limitations of the study include that participating infants were in different age groups (6–11 months) during screening, and we have no repeated measures to further assess the reliability of the measures. Moreover, the m-ADBB scorings also included the infants who were scored with the full version. Thus, some of the 45 infants who were double scored to measure interrater agreement of the modified version may also have been scored with the full version by the trained staff, which could have implications for the accuracy of scorings and be part of the explanation to the improved agreement between raters. Finally, for the m-ADBB, we do not have expert scorings to use as reference scores.

## Conclusion

The current study suggests that the modified version of the ADBB scale is an acceptable approach to achieve adequate interrater agreement in a large community-based study in Nepal. The scores suggest high levels of social withdrawal in these Nepalese infants. Based on the results, however, there are uncertainties on the internal consistency of both scales, and the validity of the scales needs to be examined in future studies. More effective training strategies for administration and additional cultural-specific instructions could be important steps to take before the scale is widely used in this context.

## Data Availability Statement

The datasets generated for this study are available on request to the corresponding author.

## Ethics Statement

The study was reviewed and approved by the Nepal Health Research Council (Reg 233/2014) and the Norwegian Regional Committee for Medical and Health Research Ethics (REC # 2014/1528). Written informed consent to participate in this study was provided by the participants’ legal guardian/next of kin.

## Author Contributions

TS, IK, and RC designed the study. RC, MU, SR, MS, and LS conducted the research and were responsible for the field implementation. MU, SR, MS, IK, and HB were responsible for scoring ADBB and standardization. MU and IK analyzed the data and interpreted the results. MU, TS, and IK had primary responsibility for the final content. All authors read and approved the final manuscript.

## Conflict of Interest

The authors declare that the research was conducted in the absence of any commercial or financial relationships that could be construed as a potential conflict of interest.
